# A Five-Gene Prognostic Nomogram Predicting Disease-Free Survival of Differentiated Thyroid Cancer

**DOI:** 10.1155/2021/5510780

**Published:** 2021-06-15

**Authors:** Pan Ruchong, Tang Haiping, Wang Xiang

**Affiliations:** ^1^Faculty of Medical Technology, Chongqing Three Gorges Medical College, Chongqing, China; ^2^Transfusion Section, Chongqing University Three Gorges Hospital, Chongqing, China

## Abstract

**Background:**

Differentiated thyroid cancer (DTC) is the most common type of thyroid tumor with a high recurrence rate. Here, we developed a nomogram to effectively predict postoperative disease-free survival (DFS) in DTC patients.

**Methods:**

The mRNA expressions and clinical data of DTC patients were downloaded from the Cancer Genome Atlas (TCGA) and the Gene Expression Omnibus (GEO) database. Seventy percent of patients were randomly selected as the training dataset, and thirty percent of patients were classified into the testing dataset. Multivariate Cox regression analysis was adopted to establish a nomogram to predict 1-year, 3-year, and 5-year DFS rate of DTC patients.

**Results:**

A five-gene signature comprised of TENM1, FN1, APOD, F12, and BTNL8 genes was established to predict the DFS rate of DTC patients. Results from the concordance index (C-index), area under curve (AUC), and calibration curve showed that both the training dataset and the testing dataset exhibited good prediction ability, and they were superior to other traditional models. The risk score and distant metastasis (M) of the five-gene signature were independent risk factors that affected DTC recurrence. A nomogram that could predict 1-year, 3-year, and 5-year DFS rate of DTC patients was established with a C-index of 0.801 (95% CI: 0.736, 0.866).

**Conclusion:**

Our study developed a prediction model based on the gene expression and clinical characteristics to predict the DFS rate of DTC patients, which may be applied to more accurately assess patient prognosis and individualized treatment.

## 1. Introduction

Differentiated thyroid cancer (DTC) is the most common tumor in the head and neck area and accounts for approximately 90% of all cases. DTC is composed of papillary thyroid carcinoma (PTC) and follicular thyroid carcinoma (FTC), which both originate from follicular cells of the thyroid [1, 2]. Although the 10-year mortality rate of DTC is only 1.7%, the postoperative recurrence rate is up to 35%, and the mortality rate of recurrent patients is 48% [3–5]. The ability to precisely predict individual risk of recurrence has become an important and effective measure to prevent the recurrence of DTC. Currently, the commonly used risk stratification includes the American Joint Committee on Cancer (AJCC) staging system，the American Thyroid Association (ATA) staging system, and the European Thyroid Association (ETA) staging system [6–8]. Although these traditional risk stratification systems are useful for predicting overall patient outcome, it is difficult to apply them for individualized and accurate prediction. Due to the emergence of molecular tumor profiling, analysis of prognostic-related genes has significantly progressed, making it possible to accurately predict the prognosis of patients at the molecular level. In previous studies of cancer prediction analysis, the prediction system integrated gene signature and clinical characteristics, which provides more accurate and reliable prediction results in contrast to the traditional staging systems [9–11].

At present, nomogram has been accepted as prognostic evaluation method based on evidence-based and precision medicine, and it has been widely used in prognosis analysis of various tumors [12–14]. In this study, we analyzed the mRNA expression of cancer and paracancerous tissue of DTC patients from the Cancer Genome Atlas (TCGA) and Gene Expression Omnibus (GEO) databases to screen for differentially expressed genes (DEGs) related to patient disease-free survival (DFS). Multivariate Cox regression analysis was employed to establish a gene signature, which was compared with the traditional and other risk stratification models. Gene signature indicating high- and low-risk groups underwent Kaplan-Meier survival correlation curve analysis and GSEA pathway enrichment analysis. Finally, a nomogram based on gene signature and clinical characteristics was plotted to predict the 1-, 3-, and 5-year DFS rate of patients with DTC.

## 2. Materials and Methods

### 2.1. Data Source and Screening of Differentially Expressed Genes

The mRNA expression data (HTSeq counts) of cancerous and paracancerous tissue were collected up to March 29, 2020 from the TCGA-THCA (https://cancergenome.nih.gov/), GSE27155, and GSE53157 datasets (CEL files). To obtain the mRNA expression from normal tissue and tumors, data from the GEO database (https://www.ncbi.nlm.nih.gov/geo/) was downloaded. The edge*R* package (version3.26.3) and limma package (version 3.38.3) in *R* (version 3.6.0) were run to eliminate very low-expression genes. The mRNA expression data obtained from TCGA and GEO was normalized to eliminate errors. Then, log (fold change) > 1 and false discovery rate (FDR) < 0.05 were set to screen DEGs in the cancer and control group. Venn diagrams were employed to determine the intersection between the three datasets to obtain DEGs in common. Gene Ontology (GO) and Kyoto Encyclopedia of Genes and Genomes (KEGG) enrichment of DEGs was conducted by using the DAVID bioinformatic resources (version 6.8; https://david.abcc.ncifcrf.gov/) with *P* < 0.05 as the selection condition. The clinical data of patients included DFS time, recurrence status, cancer type, gender, age, tumor AJCC staging, tumor T staging, tumor N staging, and tumor M staging [15]. All clinical parameters were based on the AJCC staging system (8th edition) for thyroid cancer, [16] and clinical data with missing information or duplicate samples were excluded.

### 2.2. Establishment and Validation Analysis of Gene Signature

Samples with the normalized mRNA expression data and integrated clinical data were included in the overall analysis. A total of 70% of the total number of samples were randomly selected as the training dataset [17], and the remaining 30% of samples served as the testing dataset [18] using the caret package (version 6.0-84). Data from the training dataset were analyzed by univariate Cox regression through the survival package (version 2.44-1.1), and the DEGs related to DFS were screened out using *P* < 0.05. In addition, the glmnet package (version 2.0-18) was adopted to perform Lasso regression analysis. Data was randomly simulated 1,000-fold, then crossvalidated to select DEGs with the best predictive ability. The screened DEGs were analyzed by stepwise multivariate Cox regression analysis through the survival package in *R*. Risk score and C-index of the training dataset were calculated, and a gene signature was constructed by combining the regression coefficient and mRNA expression. The gene signature was applied to the TCGA, GSE27155, and GSE53157 datasets to evaluate its ability of distinguishing between cancer and normal tissue by AUC. The DEGs of the gene signature were used for the analysis of pairing differences of the gene expression in cancerous versus paracancerous tissue. The 1-year, 3-year, and 5-year receiver operating characteristic (ROC) curves of patients in the training dataset were plotted via the risk ROC package (version 1.0.3). Then, the gene signature model formula of the training dataset was incorporated into the data of the testing and entire dataset to achieve the ROC curve and AUC values of the data model, respectively. Subsequently, the AUC value was used as a measure to compare the performance of our model with that of other models including the five-gene signature model of Wu et al. [11], AJCC stage model, ATA model, and ETA model. The comparison between the five-gene signature model of Wu et al. and our model was performed using TCGA-THCA HTSeq count dataset.

### 2.3. Risk Grouping Analysis of Gene Signature

In the training dataset, the best cutoff point for the risk score was identified using the *X*-tile software (version 3.6.1) in order to divide the data into high-risk and low-risk groups [19]. Kaplan-Meier survival curves of high- and low-risk groups were created and plotted using the *R* survival package. In addition, a risk curve, a risk scatter chart, and a risk heat map were created to visualize the data of high- and low- risk groups. Then, the best cutoff point of the risk score in the training dataset was validated using the data of the testing and entire dataset. The Kaplan-Meier survival curve was plotted to verify the discriminative performance of the cutoff point. The high- and low- risk groups in the entire dataset were included in the gene set enrichment analysis (GSEA) (version 4.0.2 for windows), and the gene annotation set c2.cp.kegg.v7.0.symbols.gmt served as a reference. To obtain the differences in biological function and pathways between high- and low- risk groups, the results were filtered and analyzed at *P* < 0.05 and FDR < 0.25 [20].

### 2.4. Establishment and Evaluation Analysis of Predictive Nomogram

Clinical data and risk score values of patients in the entire dataset were extracted and included in the subsequent analysis. A univariate and multivariate Cox regression analysis using the *R* survival package was employed to screen for independent risk factors that affected the recurrence of DTC patients. Using the rms package (version 5.1-3.1), a nomogram was plotted, which contained all independent risk factors and related clinical data. In addition, a calibration curve was established to evaluate the predictive ability of the nomogram.

### 2.5. Statistical Analysis

Statistical analysis was performed based on *R* (version 3.6.0), where categorical variables were analyzed by *χ*^2^ test or Fisher's exact test. Continuous variables were analyzed by *t*-test, and multiple groups of continuous variables were analyzed by one-way ANOVA with Bonferroni posthoc test. The difference in prognosis between high- and low-risk groups was analyzed by the log-rank test. Of each relevant factor, the hazard ratio (HR) and 95% confidence interval (CI) were calculated. Unless otherwise specified, *P* < 0.05 was considered statistically significant.

## 3. Results

### 3.1. Data Source and DEG Screening Analysis

**Figure 1:**
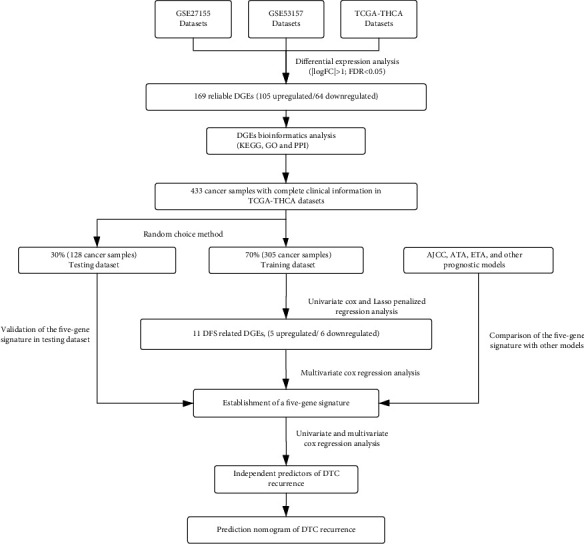
Flowchart of establishing the gene signature and prognostic nomogram of DTC patients.

**Figure 2:**
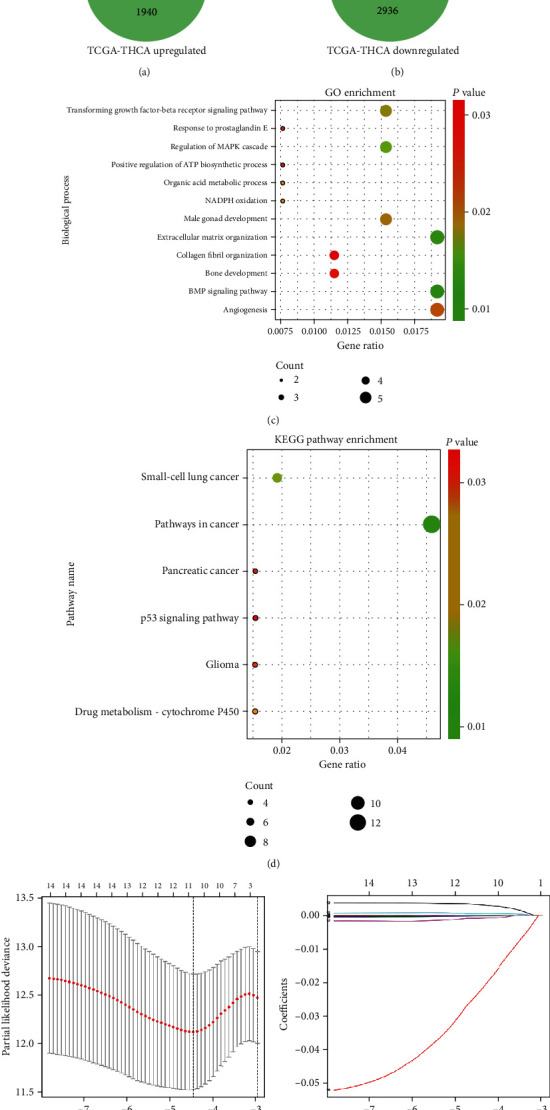
Screening of differentially expressed genes and bioinformatic analysis. (a, b) Venn diagrams show the intersection between genes differentially expressed of the three data sources. (c, d) The dot plot of the enriched biological function and KEGG pathways of the DEGs. (e, f) Lasso regression analysis of the prognostic DEGs.

The main flow of this study is presented in Figure 1.  Table 1 shows the characteristics of the three datasets containing the accession numbers of datasets, sample size, and platforms. When analyzing DEGs of the three datasets in common, 169 DEGs were obtained, including 105 upregulated genes and 64 downregulated genes (Figures 2(a) and 2(b)). GO analysis demonstrated that changes in biological processes of DEGs were mainly enriched in interactions between extracellular matrix (ECM), angiogenesis, BMP signaling pathway, transforming growth factor-beta receptor signaling pathway, and the regulation of the MAPK cascade (Figure 2(c)). Furthermore, enrichment analysis on the KEGG pathway showed that the DEGs were mainly concentrated in cancer pathways and the P53 signaling pathway (Figure 2(d)). Among the relevant clinical data of DTC patients, a total of 433 samples with complete clinical information were selected for analysis.

### 3.2. Establishment and Validation of the Five-Gene Signature

The entire dataset of the TCGA dataset was randomly divided into 305 cases in the training dataset and 128 cases in the testing dataset.  Table 2 shows the clinical baseline features of DTC patients in the training and testing datasets. In the training dataset, based on the univariate Cox analysis of DEGs and patient DFS data, it was found that a total of 14 DEGs were associated with DFS, including 8 upregulated genes and 6 downregulated genes (*P* < 0.05). For further screening through Lasso regression analysis, 11 genes were selected to perform the fitting between the mRNA and DFS data (Figures 2(e) and 2(f)). The regression coefficient and 5 DEGs obtained through multivariate Cox regression analysis and the expression of the five genes were included in the construction of the model formula to achieve the following risk score formula:

(1) $$\begin{array}{c} \text{risk score}=\left(3.12\text{E}-05\times \text{expression value of TENM}1\right)+\left(3.67\text{E}-07\times \text{expression value of FN}1\right)-\left(5.08\text{E}-04\times \text{expression value of APOD}\right)+\left(3.86\text{E}-03\times \text{expression value of F}12\right)-\left(4.76\text{E}-02\times \text{expression value of BTNL}8\right). \end{array}$$

**Figure 3:**
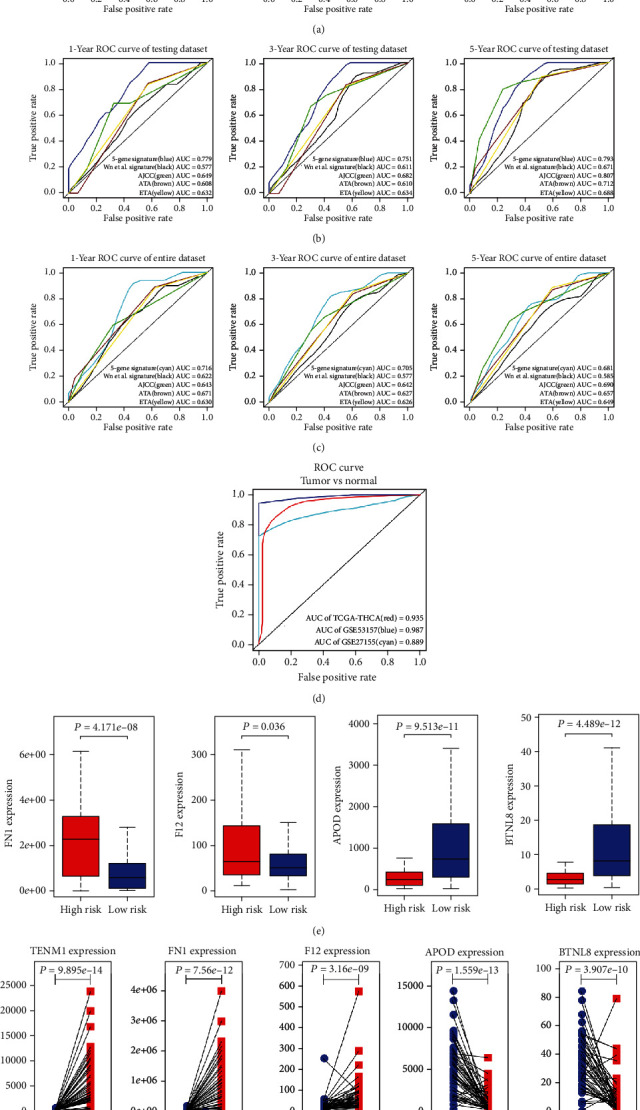
Establishment and validation of the five-gene signature. (a)–(c) ROC curves of the five-gene signature for 1-, 3-, and 5-year DTC patient recurrence compared with other models in the training, testing, and entire dataset. (d) The ability of the five-gene signature to distinguish between cancer and normal tissue in the TCGA, GSE27155, and GSE53157 datasets. (e) The difference of FN1, F12, APOD, and BTNL8 expression levels in the high- and low-risk groups of DTC recurrence patients (*P* < 0.05). (f) The pairing differences between cancerous and paracancerous tissue genes in 58 DTC patients.

In the training dataset, the C-index of the five-gene signature is 0.750 (95% CI: 0.664-0.836), and the 1-year, 3-year, and 5-year AUC were 0.686, 0.708, and 0.659, respectively. Comparison between the five-gene signature model of this study and other models shows that our model has a good predictive power within training, testing, and entire dataset (Figures 3(a)–3(c)). The ability of the five-gene signature to distinguish between cancer and normal tissue is satisfying, since the AUC of the TCGA, GSE27155, and GSE53157 datasets are 0.935, 0.889, and 0.987, respectively (Figure 3(d)). The data show that the mRNA expression levels of TENM1, FN1, and F12 are positively correlated with the risk score, while the expression levels of APOD and BTNL8 are negatively correlated with the risk score. Moreover, the expression levels of FN1, F12, APOD, and BTNL8 shows significant differences in the high- and low-risk groups of DTC recurrence (*P* < 0.05) (Figure 3(e)). In addition, according to the pairing differences between cancerous and paracancerous tissue genes in 58 DTC patients, the expression of each gene is significantly different in cancer tissue (Figure 3(f)). We found that TENM1, FN1, and F12 are highly expressed in tumor tissue, while APOD and BTNL8 have a lower expression in tumor tissue. The relationship between the expression of the five genes and clinicopathological characteristics of DTC patients are shown in supplementary figures [21].

### 3.3. Risk Grouping Analysis on the Five-Gene Signature

**Figure 4:**
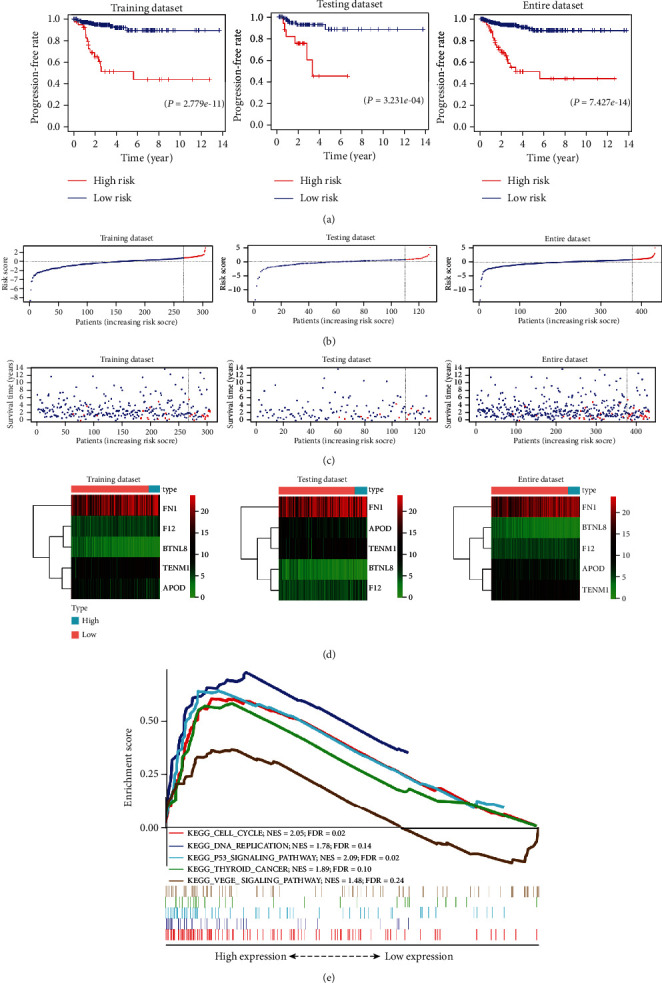
Risk grouping analysis for the five-gene signature. (a) Kaplan-Meier disease-free survival curve of high- and low-risk group in the training, testing, and entire dataset. (b)–(d) The risk curves, risk scatter plots, and risk distribution heat maps of high- and low-risk group of the training, testing, and entire dataset. (e) GSEA enrichment analysis result map of entire dataset.

The risk score was divided into high- and low-risk groups by applying the *X*-tile software (cutoff value = 0.73). Kaplan-Meier survival curves of the high- and low-risk group of the training dataset, the testing dataset, and the entire dataset were plotted (Figure 4(a)). All groups have a significant difference in DFS between the high- and low-risk groups (*P* < 0.001). The risk curves, risk scatter plots, and risk distribution heat maps of these three datasets are presented in Figures 4(b)–4(d). GSEA enrichment analysis shows that the P53 signaling pathway, VEGF signaling pathway, thyroid cancer, cell cycle, and DNA replication were more active in the high-risk group when compared to that in the low-risk group (*P* < 0.01, FDR < 0.25) (Figure 4(e)).

### 3.4. Establishment and Evaluation of a Predictive Nomogram

**Figure 5:**
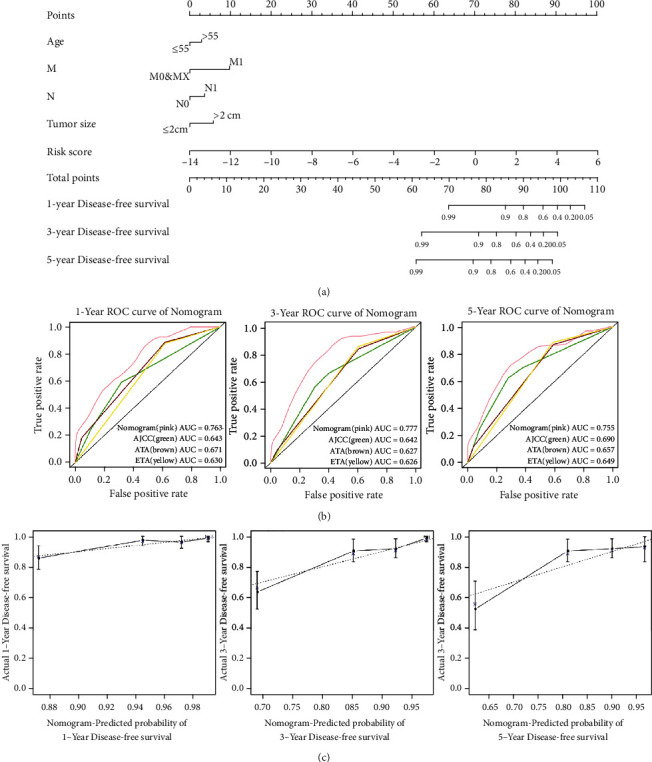
Establishment and validation of a predictive nomogram. (a) The nomogram for predicting proportion of DTC patients with 1-, 3-, and 5-year disease-free survival. (b) ROC curves of the nomogram for 1-, 3-, and 5-year DTC patients recurrence compared with other models in the entire dataset. (c) The calibration curve of the nomogram.

Univariate Cox regression analysis was performed on the risk scores of the five-gene signature combined with clinical characteristics of the entire dataset (Table 3). Our data show that age (>55 vs. ≤55, *P* = 0.004), tumor size (>2 cm vs. ≤2 cm, *P* = 0.008), N stage (N1 vs. N0, *P* = 0.048), M stage (M1 vs. M0 and MX, *P* < 0.001), AJCC stage (stages III and IV vs. stages I and II, *P* = 0.001), and risk score (*P* < 0.001) were correlated with patient recurrence. In the univariate analysis, the parameters with *P* < 0.05 were further incorporated into the multivariate Cox regression analysis. The multivariate Cox regression results indicated that risk score and M stage were independent risk factors for recurrence in DTC patients (*P* < 0.05) (Table 4). The C-index of the nomogram was 0.797 (95% CI: 0.730-0.864). The 1-year, 3-year, and 5-year ROC curves of the nomogram model were established (Figure 5(a)), and the AUC results demonstrated that the accuracy of the nomogram prediction was superior to other models (Figure 5(b)). Moreover, calibration curve analysis was performed on the nomogram model (Figure 5(c)).

## 4. Discussion

Thyroid cancer is the common endocrine malignancy that occurs in the head and neck, of which the pathogenesis is poorly understood [1]. Exposure to radiation, iodine intake, genetics, and other factors can cause thyroid cancer [1]. Even after patients have undergone standard surgery, ^131^I and thyroid hormone suppression therapy, 5%-23% of patients still suffer from metastatic recurrence [22]. A good prediction model of patient prognosis can effectively identify patients with high risk of recurrence and foster their individualized indepth treatment to achieve better therapeutic outcomes. The combined prediction of various prognostic markers derived from gene expression profiling can reflect the prognosis of individual patients at the molecular level. Such a gene signature can be complementary with traditional AJCC, ATA, and ETA staging prediction systems.

In this study, GO biological process enrichment analysis and KEGG pathway enrichment analysis were performed using cancer-related DEGs, which were derived from the intersection between the TCGA-THCA, GSE27155, and GSE53157 datasets. The results showed that transforming growth factor-beta receptor signaling pathway, BMP signaling pathway, regulation of MAPK cascade, and extracellular matrix organization are significantly enriched in the analysis of biological processes. Among the extracellular matrix proteins, collagen, fibronectin, and integrin represent the major components. Some studies indicate that type IV collagenase and matrix metalloproteinases secreted by thyroid cancer cells can promote the metastasis of cancer cells by destroying the extracellular matrix structure, which affects the prognosis of thyroid cancer [23–25]. Fibronectin and integrin can participate in the adhesion process of tumor cells and extracellular matrix through activation of the Ras/Raf/Mek pathway and the calmodulin dependent kinase-II (CaMKII) pathway [26]. Transforming growth factor-beta 1(TGF-*β*1) and BMP-2 are members of the transforming growth factor-beta family, which exert important roles in tumor growth and invasion. TGF-*β*1 and epidermal growth factor- (EGF-) like ligands have opposite roles in different stages of thyroid cancer growth: on one hand, they act as tumor suppressors through inhibiting the proliferation of thyroid cells and regulating the formation of extracellular matrix [27, 28]; on the other hand, they can promote angiogenesis in advanced stage of thyroid cancer [29]. TGF*β* and BMPs pathways can contribute to the synergistic suppression of tumor growth, but atypical TGF*β* that activates PI3K/AKT signaling can reverse this suppression and promote tumor cell proliferation [30]. The mitogen-activated protein kinase (MAPK) pathway is one of the most classical signal transduction pathways in thyroid cancer. In this pathway, BRAF-mutations and RET/PTC rearrangements can promote the transformation of thyroid follicular cells into papillary thyroid cancer, which is considered as a hallmark for PTC development and progression [31].

The five-gene signature was established as a new molecular predictive index for the recurrence in DTC patients. The results of multivariate Cox regression coefficient of the five genes indicated that TENM1, FN1, and F12 were protective factors, while APOD and BTNL8 were unfavorable elements for recurrence risk of DTC patients. Multiple studies have shown that the expression of TENM1, which acts as a cell signal transducer in neurons, is highly positively correlated with the growth and invasion of PTC, and it is a potential biomarker for early diagnosis of thyroid cancer [32–34]. FN1 encodes fibronectin and is involved in cell proliferation, adhesion, and migration. Previous studies demonstrated that FN1 is a potential therapeutic target highly related to tumor invasion in PTC and medullary carcinoma, and it is also one of the markers to distinguish malignant from benign nodules in thyroid cancer [35–37]. F12 encodes coagulation factor XII which circulates in blood as a zymogen; however, the relationship between F12 and tumorigenesis has not been reported. This study pointed out that F12 is highly expressed in DTC tissues. Furthermore, our results demonstrated that the F12 expression is higher in FTC tissues than in PTC tissues, and its expression is highly correlated with patient age, lymph node metastasis, and postoperative recurrence risk (*P* < 0.05). APOD, known as apolipoprotein D, is regulated by P73 and P63 proteins, which belong to the P53 tumor suppressor family [38]. Moreover, it has been shown that APOD can inhibit the proliferation of cancer cells in breast cancer, prostate cancer, and colorectal cancer cell lines, and it may be used as a marker of the initial stage of tumor deterioration [39–41]. Butyrophilin-like (BTNL) protein can regulate T lymphocyte response. Research indicates that BTNL8 is also related to inflammatory disorders and tumorigenesis. In intestinal tumors, where BTNL8 was found to be downregulated, it can enhance the immune response mediated by T cells and plays a role in immune surveillance of tumor cells [42].

In this study, we established a high- and low-risk group (cutoff value = 0.73) of a five-gene signature through *X*-tile software. GSEA enrichment analysis of the two groups showed that the P53 signaling pathway and the VEGF signaling pathway were significantly enriched in the high-risk group. The P53 signaling pathway plays a central role in tumorigenesis. *TP53* is one of the most important tumor suppressor genes, which has the functions of inhibiting cell proliferation, participating in cell cycle regulation and inducing cell apoptosis. Due to a short protein half-life, wild-type P53 is expressed at a low level in cells, whereas mutant (inactive) P53 is frequently overexpressed in cancer cells due to its higher stability. Therefore, the high expression of the P53 protein in the tumor tissue is closely related to poor prognosis [43]. Vascular endothelial growth factor (VEGF) is an essential factor for the development of blood vessels, which is crucial for the growth of tumors. Lymphatic vessel density and VEGF-C expression have been found to be significantly different between benign and malignant tissue [44]. The expression of VEGF is highly correlated with the tumorigenesis and the prognosis of DTC [45, 46]. The abovementioned pathway analysis showed that the biological processes related to cancer growth was enriched in the high-risk group. The Kaplan-Meier survival correlation curve of the training dataset and the testing dataset intuitively reflected the difference in DFS rates of the two groups, suggesting that the cutoff points of the high- and low-risk groups were well differentiated.

In many studies, nomograms for predicting the recurrence of thyroid cancer patients had been previously formulated. Pathak et al. established a 10-year recurrence prediction nomogram (C-index 0.76) for thyroid cancer patients [47]. Ding et al. successfully established a 3-, 5-, and 10-year cancer recurrence prediction nomogram (C-index 0.70) for PTC patients [48]. Wu et al. constructed a prognostic nomogram related to five-gene signature by analyzing the data from GEO and TCGA, with the goal to predict progression-free interval of PTC patients. The C-index of the model is 0.76, which has good predictive value for PTC [11]. However, a relevant prognostic nomogram for DTC patients has not been developed so far. In this study, we found that the risk score and M stage were independent risk factors for recurrence in DTC patients (*P* < 0.05), and we established a 3-, 5-, and 10-year cancer recurrence prediction nomogram (C-index 0.80) for DTC patients.

Although this study segmented 30% of the data from the overall population for validation, a large sample validation analysis of other cohorts could not be carried out due to the limited availability of data. Since in this study, the clinical characteristics of patients that could be included in the analysis was limited, and more indepth verification is warranted. It is worth noting that this study is the first analyzing the recurrence prediction of DTC patients from the level of the prognostic related gene expression, and its accuracy and individualized prediction ability were more prominent compared to that of traditional clinical feature prediction models.

In conclusion, we have successfully established a nomogram of a five-gene signature combined with traditional clinical characteristics to predict the 1-year, 3-year, and 5-year DFS rate of DTC patients, which can be used as a tool to assess patients' postoperative recurrence rate and to formulate an individualized, accurate treatment strategy.

## Figures and Tables

**Table 1:** Details of the datasets used for screening differentially expressed genes.

Datasets	Platform	Sample size (tumor/control)
GSE27155	Affymetrix Human Genome U133A Array	Tumor:64/control:4
GSE53157	Affymetrix Human Genome U133 Plus 2.0 Array	Tumor:11/control:3
TCGA-THCA	Illumina RNA Sequencing	Tumor:509/control:58

**Table 2:** Clinical baseline features of DTC patients in the training and testing datasets.

Clinical features	Training dataset 305		Testing dataset 128	
	*n*	%	*n*	%
Cancer type, *n* (%)				
PTC	247	80.98	104	81.25
FTC	58	19.02	24	18.75
Age, *n* (%)				
≤55	223	73.11	88	68.75
>55	82	26.89	40	31.25
Gender, *n* (%)				
Male	79	25.90	39	30.47
Female	226	74.10	89	69.53
Disease-free survival (year) (mean ± SD)	3.48 ± 2.84		2.85 ± 2.51	
Recurrence, *n* (%)				
Yes	31	10.16	13	10.16
No	274	89.84	115	89.84
Tumor size, *n* (%)				
≤2 cm	100	32.79	28	21.88
>2 cm	205	67.21	100	78.12
N, *n* (%)				
N0	153	50.16	70	54.69
N1	39	12.79	16	12.50
N1a	65	21.31	20	15.63
N1b	48	15.74	22	17.18
M, *n* (%)				
M0	182	59.67	79	61.72
M1	5	1.64	2	1.56
MX	118	38.69	47	36.72
Anatomic site, *n* (%)				
Bilateral	55	18.03	28	21.88
Isthmus	13	4.26	6	4.69
Unilateral	237	77.71	94	73.43
Stage, *n* (%)				
Stage I	177	58.03	70	54.69
Stage II	28	9.18	14	10.94
Stage III	68	22.30	27	21.09
Stage IV	32	10.49	17	13.28
ATA risk stratification, *n* (%)				
High risk	14	4.59	8	6.25
Intermediate risk	184	60.33	67	52.34
Low risk	107	35.08	53	41.41
ETA risk stratification, *n* (%)				
High risk	198	64.92	75	58.59
Low risk	100	32.79	48	37.50
Very low risk	7	2.29	5	3.91

**Table 3:** Univariate Cox regression analysis.

Clinical features	Statistics	RR (95% CI)	*P* value
Risk score (mean ± SD)	−0.396 ± 1.471	2.436 (1.900-3.123)	<0.001
Cancer type, *n* (%)			
PTC	351	1	
FTC	82	0.713 (0.301-1.687)	0.441
Age, *n* (%)			
≤55	311	1	
>55	122	2.394 (1.320-4.341)	0.004
Gender, *n* (%)			
Male	118	1	
Female	315	0.772 (0.409-1.456)	0.423
Tumor size, *n* (%)			
≤2 cm	128	1	
>2 cm	305	4.046 (1.446-11.317)	0.008
N stage, *n* (%)			
N0	223	1	
N1	210	1.856 (1.004-3.431)	0.048
M stage, *n* (%)			
M0 and MX	426	1	
M1	7	6.692 (2.375-18.853)	<0.001
AJCC stage, *n* (%)			
Stages I and II	289	1	
Stages III and IV	144	2.746 (1.515-4.977)	0.001
Anatomic site, *n* (%)			
Unilateral	331	1	
Isthmus	19	0.469 (0.064-3.427)	0.455
Bilateral	83	1.258 (0.599-2.641)	0.544

**Table 4:** Multivariate Cox regression analysis.

Clinical features	Nonadjusted		Adjust I		Adjust II	
	RR (95% CI)	*P* value	RR (95% CI)	*P* value	RR (95% CI)	*P* value
Risk score (mean ± SD)	2.171 (1.626-2.898)	<0.001	2.148 (1.620-2.849)	<0.0001	2.169 (1.636-2.874)	<0.0001
Cancer type, *n* (%)						
PTC	1		NA	NA	NA	NA
FTC	1.000 (0.392-2.552)	1.000				
Age, *n* (%)						
≤55	1		1		1	
>55	1.367 (0.579-3.228)	0.476	1.336 (0.580-3.079)	0.496	1.584(0.811-3.093)	0.178
Gender, *n* (%)						
Male	1		NA	NA	NA	NA
Female	1.231 (0.619-2.450)	0.553				
Tumor size, *n* (%)						
≤2 cm	1		1		1	
>2 cm	2.306 (0.784-6.784)	0.129	2.339 (0.804-6.804)	0.119	2.444 (0.847-7.054)	0.098
N, *n* (%)						
N0	1		1		1	
N1	1.673 (0.837-3.344)	0.145	1.644 (0.860-3.143)	0.133	1.739 (0.927-3.261)	0.085
M, *n* (%)						
M0 and MX	1		1		1	
M1	4.576 (1.523-13.755)	0.0274	4.411 (1.521-12.788)	0.006	4.478 (1.546-12.967)	0.006
Stage, *n* (%)						
Stages I and II	1		1		NA	NA
Stages III and IV	1.287 (0.569-2.908)	0.545	1.313 (0.586-2.943)	0.508		
Anatomic site, *n* (%)						
Unilateral lobe	1		NA	NA	NA	NA
Isthmus	0.689 (0.091-5.183)	0.717				
Bilateral lobe	1.354 (0.628-2.916)	0.439				

Adjust I model adjusts for parameters with *P* value <0.05 based on univariate Cox regression analysis. Adjust II model adjusts for risk score, age, tumor size, N, and M.

## Data Availability

The data used to support the findings of this study have been deposited in the figshare repository: (doi:10.6084/m9.figshare.11734497.v2; doi:10.6084/m9.figshare.11734500.v2; doi:10.6084/m9.figshare.11734494.v2).
